# Research on the Measurement Technology for Pretension Stress on Small-Sized Bolts Based on the Piezoelectric Ultrasonic Resonance Method

**DOI:** 10.3390/ma17235802

**Published:** 2024-11-26

**Authors:** Bing Chen, Chunlang Luo, Li Xia, Lintao Xu, Guanglong Yan, Feifei Qiu, Guoqing Gou

**Affiliations:** 1 Key Laboratory of Advanced Technologies of Materials, Ministry of Education, School of Materials Science and Engineering, Southwest Jiaotong University, Chengdu 610031, China; 2 Zhejiang Academy of Special Equipment Science, Hangzhou 310009, China

**Keywords:** small-sized bolts, bolt pretension, ultrasonic time delay methods, ultrasonic resonance method

## Abstract

With the widespread application of small-sized bolts in aerospace and other fields, the demand for measuring their connection structures is increasing. Currently, although ultrasonic longitudinal wave methods are commonly used for bolt pretension stress measurement, their accuracy is limited for small-sized bolts. This paper proposes a piezoelectric acoustic resonance method (PZTAR) for small-sized bolt pretension stress measurement based on acoustic elasticity theory, ultrasonic resonance principles, and a bolt stress–strain model. The method involves analyzing the ultrasonic time-domain signals of small-sized bolts under load in the frequency domain to better evaluate the changes in the ultrasonic frequencies under different pretension stress. The effectiveness of this method is verified through pretension stress measurement experiments. The results indicate that the proposed ultrasonic resonance method achieves an average error of less than 5% for M5 specification bolts. Compared to traditional ultrasonic time delay methods, the proposed method demonstrates higher measurement accuracy. Additionally, the ultrasonic resonance method exhibits better robustness during the measurement process.

## 1. Introduction

The pretension of bolts plays a decisive role in the critical performance indicators of the connected components, such as the static and dynamic characteristics, seismic resistance, and structural stability [1,2,3]. Therefore, accurately measuring bolt pretension is of paramount importance. Currently, the commonly used methods for measuring bolt pretension include the torque wrench method [3,4], resistance strain gauge method [5,6], photoelasticity method [7,8], piezoelectric impedance method [9,10], fiber Bragg grating method [11,12], and ultrasonic method [13,14]. The torque wrench method can only estimate pretension indirectly through torque. The strain gauge method, which requires attaching strain gauges, is suitable only for large-sized, non-full-thread bolts. The fiber Bragg grating method involves expensive equipment and requires skilled operation. The ultrasonic method, owing to its advantages of being non-destructive, rapid, and highly accurate, has been widely applied in bolt pretension measurement.

The commonly used ultrasonic method for measuring bolt pretension primarily involves utilizing the ultrasonic time delay method in combination with the acoustoelastic effect to test bolt pretension. The acoustoelastic effect mainly describes how the propagation speed of ultrasonic waves in an elastic material changes when the internal static stress field of the material changes [15]. Therefore, when the pretension of a bolt changes, the propagation speed of the ultrasonic waves within the bolt also changes. Additionally, the bolt itself undergoes a slight axial strain, corresponding to the applied force. As a result, the propagation time of the ultrasonic waves inside the bolt varies under different pretensions. By measuring the time differences under various pretension conditions, the bolt pretension can be calculated [16,17,18,19].

Both domestic and international scholars have made significant contributions to the ultrasonic measurement of bolt pretension. Lee et al. [20] conducted experiments on bolts of specifications M8, M10, M12, and M14 by establishing a mathematical relationship between mechanical deformation and thermal deformation, proposing a calibration equation applicable to various types of bolts. Chaki et al. [21] suggested using the ratio of the propagation speeds of ultrasonic shear and longitudinal waves in bolts under different loading conditions as a characteristic parameter for detecting bolt pretension. This method eliminates the need for traditional single-wave methods to measure the ultrasonic transit time in an unloaded state, and experiments on M20 bolts over 200 mm in length showed an accuracy of approximately 90%. Pan et al. [22] proposed a pretension measurement method based on the combination of shear waves and longitudinal ultrasonic waves, and they verified the accuracy on M16 × 140 and M20 × 140 bolts, achieving a relative error within 5%. Kim et al. [23] introduced an ultrasonic technique for stress measurement using mode conversion. By analyzing the effect of axial stress on the ultrasonic wave speed in axially symmetric cylindrical solids and establishing a linear acoustoelastic equation, experiments on M22 × 134 and M22 × 160 bolts resulted in a measurement error of less than 5%.

From these studies, it is evident that current research mainly focuses on bolts of M10 specification and above, with a length-to-diameter ratio greater than 3:1. For smaller bolts, the measurement of assembly pretension using the ultrasonic time delay method presents several challenges. Due to size constraints, only smaller diameter piezoelectric transducers can be used, significantly reducing the signal strength of the ultrasonic waves. Additionally, reflection signals from the threads interfere with those from the bolt’s bottom, further reducing the signal-to-noise ratio and complicating the extraction of effective signals. Moreover, the minimal time variation caused by stress in small-sized bolts requires equipment with high acquisition resolution, which is difficult to achieve with conventional ultrasonic time delay measurement devices due to hardware limitations. Consequently, research on the pretension measurement technology for small-sized bolts using the ultrasonic time delay method is relatively challenging.

Ultrasonic resonance technology is a method that combines electromagnetic ultrasonic or piezoelectric ultrasonic techniques with resonance thickness measurement technology. It leverages the characteristics of ultrasonic wave propagation in materials and uses resonance phenomena to evaluate the material thickness and micro-damage. This technology is often used in the thickness measurement of thin materials, material quality assessment, and defect detection, offering advantages such as speed, accuracy, and non-destructiveness. Rahammer et al. [24] improved the activation and detection efficiency of low-energy vibration thermography by periodically scanning ultrasonic excitation frequencies and performing Fourier transforms on temperature data at modulation frequencies. Diguet et al. [25] used handmade flat coils based on electromagnetic resonance technology to measure the thickness reduction of steel plates in corrosive environments, demonstrating high measurement accuracy and consistency with the actual thickness reduction. Cai et al. [26] proposed an electromagnetic acoustic resonance (EMAR) mobile scanning identification method based on the frequency–frequency energy density precipitation (FFEDP) algorithm to address the poor performance of traditional ultrasonic thickness measurement methods in detecting stepped thickness changes in test samples. This method accurately extracted the thickness information of stepped samples with millimeter-level resolution. Li et al. [27] combined EMAT with the ultrasonic resonance method to address the low energy conversion efficiency of electromagnetic ultrasonic transducers, proposing a nonlinear EMAR technique to evaluate thermal damage in metal materials, providing sufficiently high signal amplitudes for generating higher harmonics. Currently, research on ultrasonic resonance mainly focuses on the field of electromagnetic ultrasonics, primarily for defect and thickness detection of samples. However, the use of piezoelectric ultrasonic resonance to measure bolt pretension requires further exploration.

The main purpose of this study was to propose a piezoelectric acoustic resonance method (PZTAR) for measuring the pretension stress of small-sized bolts. The main contributions of this paper can be summarized as follows. Based on the acoustoelastic effect and ultrasonic resonance theory, the theory of small-sized bolt pretension measurement by PZTAR was established. Additionally, this work developed an ultrasonic resonance testing device suitable for detecting the pretension stress of small-sized bolts under tensile deformation. By varying the external load, the frequency shift changes were observed. The measurement accuracy of PZTAR was verified by taking the loading stress of the tensile testing machine as the actual pretension stress of the bolt. Comparisons of the PZTAR measured results with ultrasonic time delay measured results showed that this testing device is highly feasible. This provides technical support for future the pretension stress of small-sized bolt detection.

## 2. Measurement Theory of PZTAR

### 2.1. Acoustoelastic Theory

The propagation speed of elastic waves in stressed solid materials depends not only on the material’s second-order elastic constants and density but also on its higher-order elastic constants and stress, manifesting as the acoustoelastic effect. The acoustoelastic theory studies the relationship between the propagation speed of elastic waves and stress, forming one of the theoretical bases for stress measurement using ultrasonic methods [3,28,29].

The assumptions underlying acoustoelastic theory include the following. (1) The object is hyperelastic and homogeneous. (2) The solid adheres to the continuity assumption. (3) Small amplitude wave perturbations are superimposed on the static finite deformation of the object. (4) The deformation process of the object is assumed to be isothermal or isentropic.

When longitudinal waves propagate parallel to the stress direction in bolts, the change in the longitudinal wave velocity is closely related to stress, material density, and second- and third-order elastic constants, which can be expressed as [30]:

(1) $$V^{2}=C^{2}-\left[\left(\gamma +2\mu \right)\left(4\gamma +10\mu +4m\right)/\mu +\gamma +2J\right]\cdot \sigma /\left[\rho_{0}\cdot \left(3\gamma +2\mu \right)\right]$$

where *V* represents the sound velocity of the p-wave along the stress direction; *C* represents the longitudinal wave sound velocity of the bolt without pretension.γ, μ are the second-order elastic constants, namely the Lame constant;J, m represent the third order elastic constant, namely the Murnaghan constant; σ indicates the magnitude of the stress; and $\rho_{0}$ indicates the material density.

### 2.2. Ultrasonic Resonance Theory

**Figure 1:**
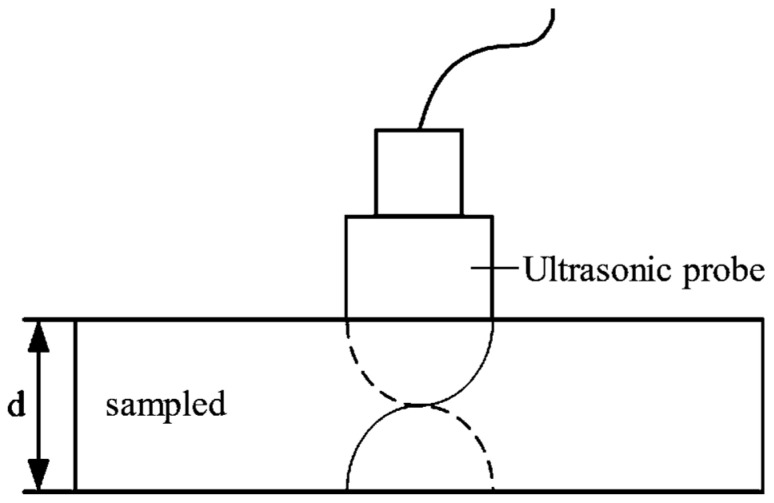
Schematic diagram of ultrasonic resonance thickness measurement.

The ultrasonic transducer is stimulated by a continuous electrical signal to emit a continuous ultrasonic wave. After the ultrasonic wave enters the material to be measured, it will be reflected at the bottom interface of the material. When there is mutual interference between the incident wave and the reflected wave, if the reflection has a total reflection phenomenon at a certain frequency, then the mutual interference will lead to the formation of standing waves, which is called resonance, and the resonant thickness measurement method [31] is based on this principle. Its schematic diagram is shown in Figure 1.

According to the above analysis, the wavelength conditions for generating resonance can be obtained:

(2) nλ=2d

where n is a positive integer, representing the resonance order; λ represents the wavelength; and d is the thickness of the sample.

After sorting, the frequency conditions for generating resonance are obtained:

(3) $$d=n\frac{\lambda }{2}=\frac{nv}{2f_{n}}$$

where $f_{n}$ represents the n order resonant frequency, and v represents the ultrasonic sound speed in the sample.

However, the thickness is usually not calculated by Equation (3), because the order of the resonant frequency cannot be intuitively obtained, so usually the following formula is used to calculate the thickness:

(4) $$d=\frac{nv}{2f_{n}}=\frac{v}{2f_{1}}=\frac{v}{2\Delta f}$$

(5) $$\Delta f=f_{n+1}-f_{n}$$

where, $f_{1}$ represents the first-order resonant frequency or resonant fundamental frequency, Δf represents the resonant frequency interval.

**Figure 2:**
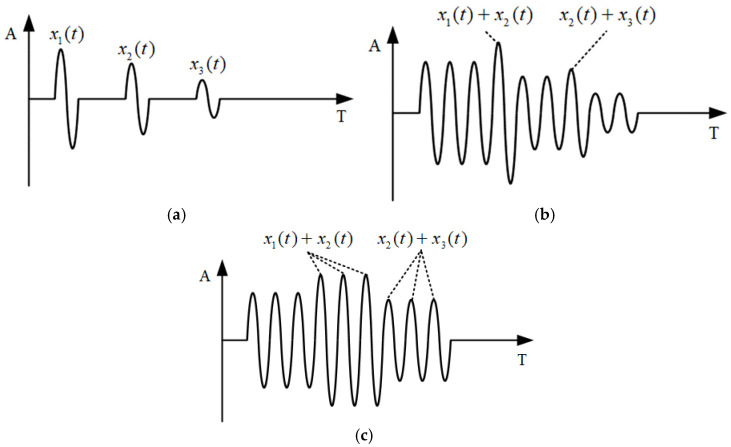
Effect of the transmitting signal period number on the resonant signal: (**a**) 1 cycle excitation; (**b**) 4 cycles excitation; and (**c**) 6 cycles excitation.

In addition, the number of cycles in the ultrasonic emission signal is crucial for generating resonance phenomena. For resonance to occur, the emitted signal must interact with the echo signal, which requires the emitted signal to be sufficiently long to influence the echo signal. Taking the third-order resonance as an example, the distribution of the emission signal and the received signal is shown in Figure 2 under different transmission signal periods. Set the emission signal as $x_{1}(t)$, and the first and second echo signals as $x_{2}(t)$ and $x_{3}(t)$. When the period of the emission signal is 1, as shown in Figure 2a, the emission signal and the echo signal are independent of each other, and no interference occurs, so resonance phenomena cannot be generated. The number of transmitting signal cycles is increased to 4, as shown in Figure 2b. At this time, the last cycle of the transmitting signal waveform interferes with the first cycle of the first echo waveform, and the last cycle of the first echo waveform interferes with the first cycle of the second echo waveform, and a resonance phenomenon occurs.

When the period of the emission signal continues to increase to 6, as shown in Figure 2c, the interference between the emission signal and the primary echo, the primary echo and the secondary echo is completely aliased, and the phenomenon of complete resonance is present. As the number of echoes increases, the high-order echo signal will decay and approximately disappear. When the period number of the emission signal exceeds 6, the ultrasonic signal will always be in a state of complete resonance, but when the period number continues to increase, the echo aliasing has a amplitude limit.

During resonance, the aliasing amplitude limit of echoes can be expressed by the following equation [32]:

(6) $$u_{\max}=\frac{u_{0}}{1-e^{-\alpha_{2d}}}$$

where $u_{0}$ represents the amplitude of the emission signal, $u_{\max}$ represents the limit amplitude, and $\alpha_{2d}$ represents the attenuation coefficient of the ultrasonic round trip in the thickness direction.

This shows that the generation of the ultrasonic resonance signal is not only related to the frequency of resonance but also to the number of cycles in the emission signal. If the number of cycles is too short, the resonance signal cannot meet the experimental requirements. Conversely, if the number of cycles is too long, it will increase the detection time for each measurement and have little effect on enhancing the amplitude of the resonance signal.

### 2.3. Measurement Principle of Bolt Pretension by PZTAR

**Figure 3:**
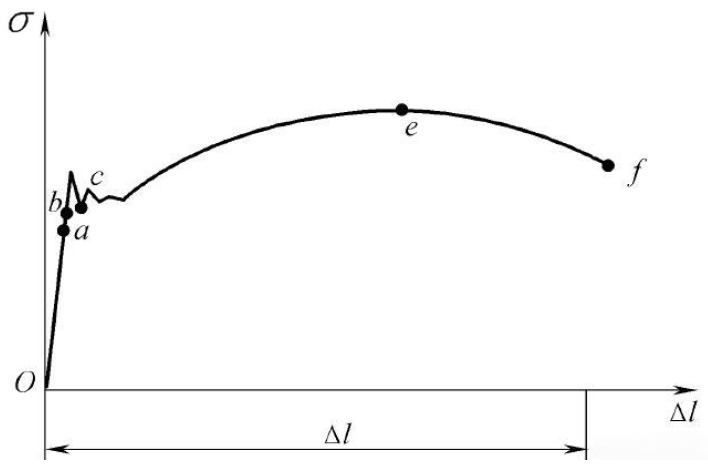
Stress–strain model of a bolt.

As the allowable range of pretension borne by the bolt is in the elastic stage, it can be seen from the stress–strain model of the bolt (Figure 3) that when the pretension is lower than the yielding stress $\sigma_{s}$ (point b in the Figure 3), the pretension σ is proportional to the strain ε and meets the following requirements:

(7) σ=E×ε

(8) $$\varepsilon =\frac{\Delta L}{L_{0}}$$

where *E* is a constant, which is called the elastic modulus or Young’s modulus.

When the bolt deforms under the pretension stress σ, the resonant thickness measurement Equation (4) is applied to the bolt measurement, and the bolt length change can be expressed as:

(9) $$\Delta L=L_{\sigma }-L_{0}=\frac{nv_{\sigma }}{2f_{\sigma }}-\frac{nv_{0}}{2f_{0}}$$

where $v_{\sigma }$ and $v_{0}$ represent the longitudinal wave sound velocity of the bolt under pretension and zero stress, respectively, and $f_{\sigma }$ and $f_{0}$ represent the *n*-th resonance frequency of the bolt under pretension and zero stress, respectively.

In small-sized bolts, the change in sound velocity caused by the pretensioning stress is very weak, so take $v_{\sigma }$ as $v_{0}$, and Equation (9) is simplified as follows:

(10) $$\Delta L=\frac{nv_{0}}{2}(\frac{1}{f_{\sigma }}-\frac{1}{f_{0}})=\frac{nv_{0}}{2}(\frac{1}{f_{0}+\Delta f_{0}}-\frac{1}{f_{0}})=-\frac{nv_{0}}{2f_{0}}\times \frac{\Delta f_{0}}{f_{0}+\Delta f_{0}}$$

where $\Delta f_{0}$ represents the change in the *n*-TH order resonant frequency when the bolt length changes.

As the bolts used in this paper are small-sized bolts, the experiment determined that $\Delta f_{0}$ is very small. Taking the 6.8 grade M5 specification bolt as an example, when $f_{0}$ is 10 MHz, Δf is about 0.05 MHz at the maximum stress of 280 MPa, which can be ignored compared with $f_{0}$. Therefore, Equation (10) can be simplified as follows:

(11) $$\Delta L=-\frac{nv_{0}}{2f_{0}}\times \frac{\Delta f_{0}}{f_{0}+\Delta f_{0}}\approx -\frac{nv_{0}}{2f_{0}}\times \frac{\Delta f_{0}}{f_{0}}=-\frac{nv_{0}\Delta f_{0}}{2f_{0}^{2}}$$

Equations (7), (8) and (11) are obtained simultaneously:

(12) $$\sigma =E\cdot \frac{\Delta L}{L_{0}}=-\frac{nv_{0}E}{2L_{0}f_{0}^{2}}\times \Delta f_{0}$$

Since the parameters n, $v_{0}$, E, $L_{0}$, and $f_{0}$ are related to the material properties, the frequency shift coefficient $K_{R}$ is defined to satisfy:

(13) $$K_{R}=\frac{nv_{0}E}{2L_{0}f_{0}^{2}}$$

The formula for measuring the bolt pre-tightening stress can be simplified to:

(14) $$\sigma =-K_{R}\Delta f_{0}=K_{R}(f_{0}-f_{\sigma })$$

**Figure 4:**
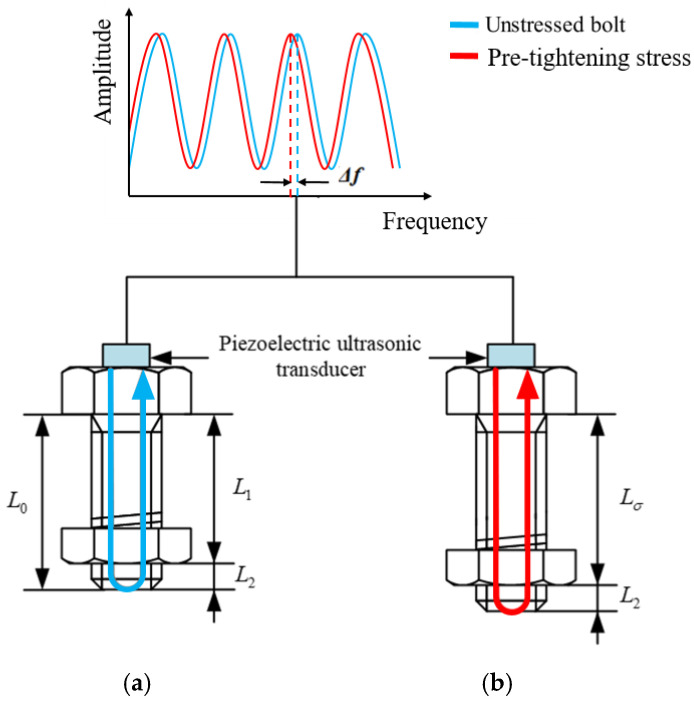
Measurement model of bolt pretension by the ultrasonic resonance method: (**a**) unstressed bolt; and (**b**) bolt after loading pretension.

According to the formula, we can observe that under the same resonance order, the ultrasonic resonance frequency will gradually decrease with the increase of the pretension stress. The measurement model is shown in Figure 4. Under the state of pretension stress and zero stress, the frequency difference of the *n*-th ultrasonic resonance $\left(f_{0}-f_{\sigma }\right)$ is the frequency shift change Δf on the resonance spectrum diagram. Therefore, this formula indicates an approximately linear relationship between the bolt pretension stress F and the ultrasonic resonance frequency shift Δf within the elastic range.

## 3. Measurement by PZTAR

### 3.1. Experimental Testing Equipment

**Figure 5:**
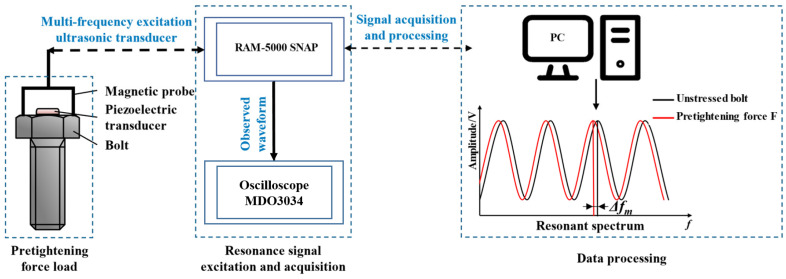
Overall structure diagram of the experimental system.

Starting from the measurement principle, the key aspects of the experimental system include precisely controlling the pretension applied to the bolt, accurately exciting and acquiring the ultrasonic resonance signals, and effectively processing the acquired signals. Therefore, the experimental system is mainly composed of three parts: the pretension application section, the ultrasonic resonance signal excitation and acquisition section, and the data processing section. The overall structure of the experimental system is illustrated in Figure 5.

**Figure 6:**
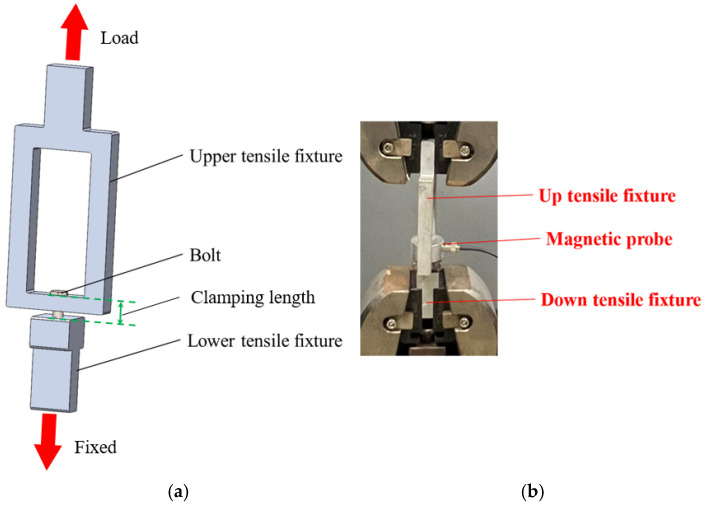
Bolt tension clamp: (**a**) schematic diagram; and (**b**) physical image.

The pretension is carried out by the tensile testing machine. Since this study is aimed at small-sized bolts, the tensile testing machine’s fixtures cannot directly apply pretension to the bolts. Therefore, specialized fixtures need to be designed to meet the experimental requirements. The three-dimensional schematic diagram of the fixtures is shown in Figure 6. The upper tensile fixture is 117.5 mm in length, 50 mm in width, and 10 mm in thickness. The clamping section is 30 mm long and 20 mm wide, designed in a U-shaped structure to facilitate the installation of bolts and magnetic transducers. The lower tensile fixture is 45 mm in length and 20 mm in width, with the threaded part being 20 mm thick. The clamping section is 30 mm long, 20 mm wide, and 10 mm thick. The diameter and depth of the threaded hole can be customized according to the size of the bolts being measured. Additionally, to avoid stress concentration at the corners, the corners are rounded.

The bolt tension clamp is shown in Figure 6, the experimental equipment is shown in Table 1, the bolt samples are all 6.8, and their parameters are shown in Table 2.

### 3.2. Frequency Shift Coefficient Calibration

According to Equation (14), $K_{R}$ cannot be obtained by calculation and needs to be obtained by fitting through calibration experiments. In order to ensure that the piezoelectric transducer can generate a sufficient number of excitation waves, the parameters of the RITEC experimental system need to be set. The fixed parameter settings are shown in Table 3. The choice of the integration gate position can affect the amplitude of the amplitude–frequency curve but has little impact on the resonance frequency position. Therefore, when performing integration processing, the blind zone following the excitation signal should be avoided as much as possible. The parameter settings for different bolt specifications are shown in Table 4.

**Figure 7:**
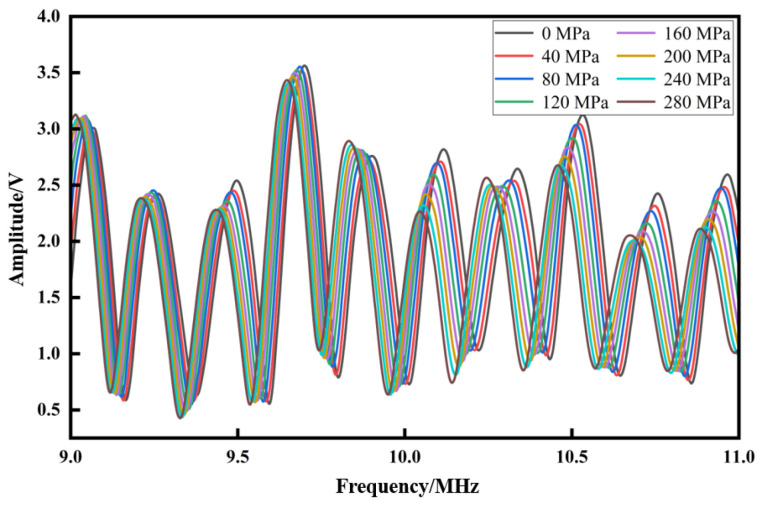
The resonance spectrum of specimen A under different stresses in the first test.

Three samples are stretched on the tensile testing machine with different clamping lengths to apply axial pretension ranging from 0 to 280 MPa, with a step control of 40 MPa. Figure 7 is the resonance spectrum of specimen A under different stresses in the first test. We can find the shift in the resonance spectrum as a whole as the stress increases. This phenomenon is consistent with the derivation in Section 2.3.

A linear function is used for fitting, and the slope value of the function is $K_{R}$. To reduce the impact of random errors, five bolts of each sample type are selected for calibration, and the average value is taken as the calibration result for that sample type, as shown in Table 5.

### 3.3. Accuracy Verification

The experimental system shown in Figure 7 is used to validate the proposed method for measuring the pretension of bolts of different specifications. The measurement experiment follows the same procedure as the calibration experiment, using bolts of the same specifications loaded on the tensile testing machine for ultrasonic resonance measurement. The loading stress from the tensile testing machine is taken as the actual pretension of the bolts. Each bolt specification is measured three times, and the results are shown in Table 6, Table 7 and Table 8.

The relative measurement error Δ of the bolt pretension determined by the ultrasonic resonance method proposed in this paper can be expressed by the following formula:

(15) $$\Delta =\frac{\left|\sigma_{1}-\sigma_{2}\right|}{\sigma_{1}}\times 100\%$$

where $\sigma_{1}$ is the stress shown by the tensile testing machine, and $\sigma_{2}$ is the pretension value measured by the ultrasonic resonance method.

The average value of the relative error of the three bolts is taken, and the results are shown in Table 9. The maximum measurement error of the proposed method is about 17.18%, and the average measurement error is less than 5%.

## 4. Measurement by Ultrasonic Time Delay Methods

### 4.1. Principle of Ultrasonic Time Delay Method

**Figure 8:**
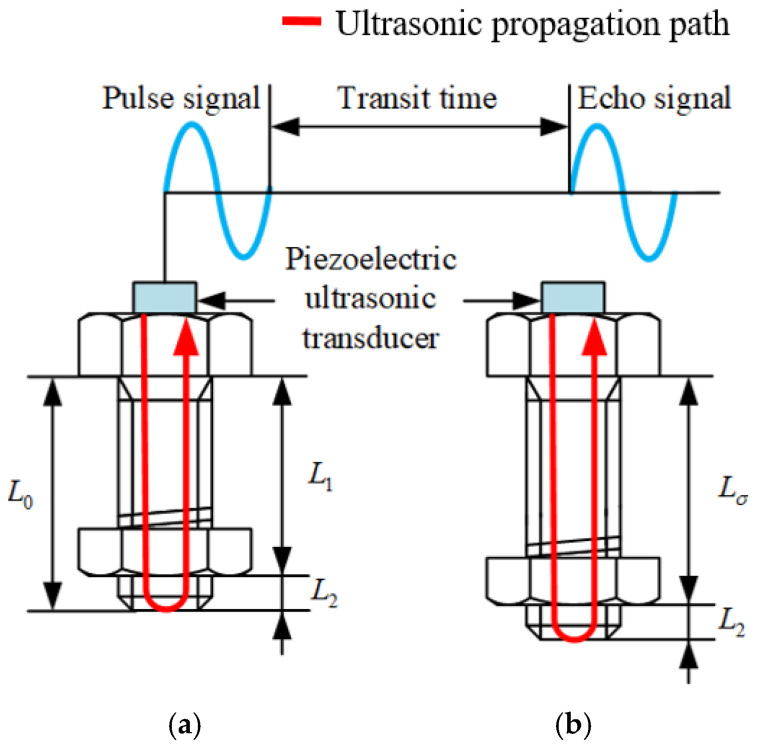
Schematic diagram of the ultrasonic travel time measurement: (**a**) unstressed bolt; and (**b**) load pretension bolt.

According to the acoustoelastic effect, the propagation speed of ultrasonic waves in a bolt changes when the bolt’s pretension changes [19,20,21,22]. Additionally, the bolt itself experiences a small axial strain corresponding to the applied stress. Therefore, the propagation time of ultrasonic waves within the bolt varies under different pretension conditions. By measuring the time difference under different pretensions, the pretension of the bolt can be calculated. The simplified formula is:

(16) $$\sigma =K_{L}\left(t_{\sigma }-t_{0}\right)$$

where *F* represents the bolt pretension; $K_{L}$ represents the pretension coefficient, which depends on factors such as the bolt’s material, size, and grip length, and is not easily measured directly, being usually obtained through calibration experiments; and $t_{\sigma }$ and $t_{0}$ represent the ultrasonic time-of-flight under stressed and unstressed conditions, respectively. The measurement of the ultrasonic time-of-flight is illustrated in Figure 8. Since the ultrasonic time-of-flight within the bolt is typically in the nanosecond range, expensive high-sampling-rate equipment is required for the time measurement.

### 4.2. Pretension Coefficient Calibration

The selection and attachment of the piezoelectric transducer to the bolt follow the same principles as the resonance method and use the same experimental platform. The parameters of the RITEC experimental system need to modify the emission signal into a pulse signal. The pretension applied to the bolt by the tensile testing machine is consistent with the resonance method. Ultrasonic signals are collected using an oscilloscope at intervals of 40 MPa. The cross-correlation algorithm is then used to calculate the waveform, obtaining the ultrasonic time delay under different pretensions. For calibration, five samples of each type are selected, and the calibration results are fitted. The calibration results are shown in Table 10.

### 4.3. Accuracy Verification

The process of the measurement experiment and calibration experiment is the same. A tensile testing machine is used to measure the pretension stress of three bolts for three kinds of samples, respectively. The measurement results are shown in Table 11, Table 12 and Table 13.

The average value of the relative error of the three bolts is taken, and the results are shown in Table 14. The maximum measurement error of time delay method is about 32.38%, and the highest average relative error is 13.75%.

## 5. Method Comparison

### 5.1. Ultrasonic Signal Comparison

**Figure 9:**
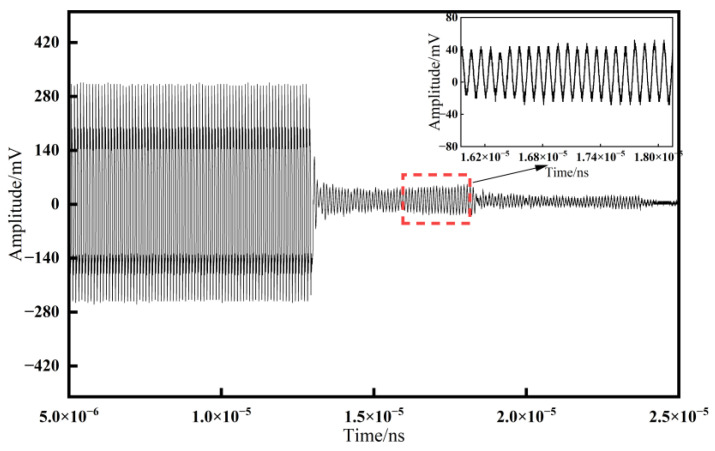
Ultrasonic signal by the resonance method.

**Figure 10:**
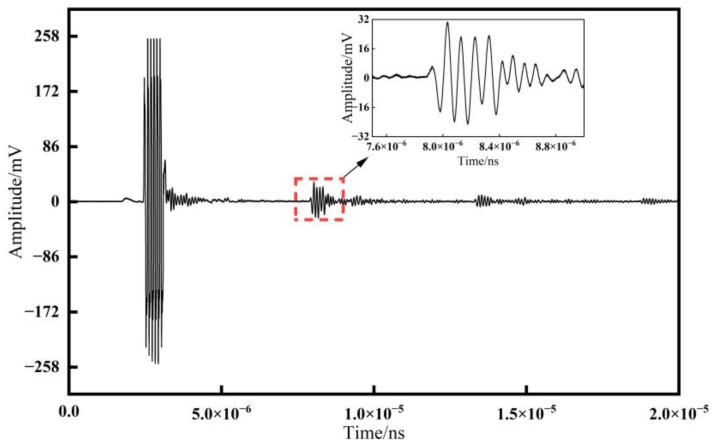
Ultrasonic signal by the time delay method.

Comparing the ultrasonic signals obtained using the resonance method and the time delay method, as shown in Figure 9 and Figure 10, we can observe the following: the amplitude of the ultrasonic signal for the resonance method is approximately 45 mV, while for the time delay method, it is about 30 mV. A higher amplitude indicates a higher signal-to-noise ratio (SNR) for the ultrasonic signal, which facilitates the extraction of effective signals and, to some extent, improves the measurement accuracy.

### 5.2. Fitting Curve Comparison

**Figure 11:**
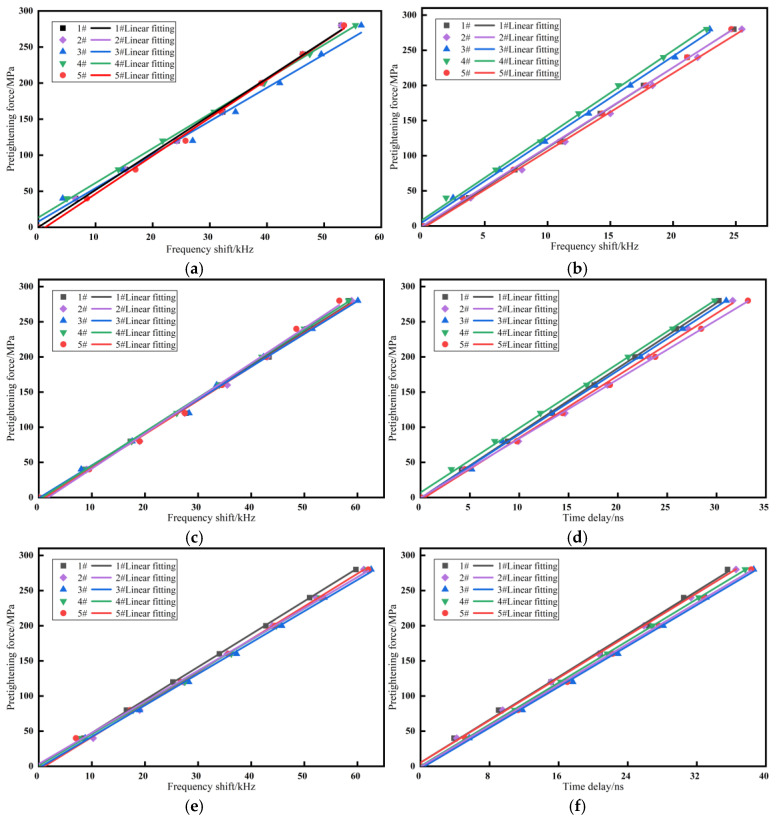
Bolt stress coefficient calibration curves: (**a**) sample A by resonance method; (**b**) sample B by resonance method; (**c**) sample C by resonance method; (**d**) sample A by time delay method; (**e**) sample B by time delay method; and (**f**) sample C by time delay method.

Comparing the calibration curves of the two methods, as shown in Figure 11, it is evident that the resonance method exhibits good repeatability and consistency across all three bolt specifications. In contrast, the time delay method shows poor repeatability and consistency for the M5 × 10 bolts. However, as the bolt length increases, the consistency and repeatability of its calibration curve improve. This indicates that in the time delay method, the bolt size becomes a limiting factor for measurement accuracy, leading to significant measurement errors for small-sized bolts.

The time delay method uses an oscilloscope for ultrasonic signal acquisition, with a sampling rate of 2.5 GSPS, corresponding to a time interval of 0.4 ns. Based on the previous calibration results, the stress coefficient for the M5 × 10 bolts using the time delay method is 11.53 MPa/ns, giving the acquisition system a stress resolution of 4.61 MPa. The resonance method’s acquisition rate is adjusted by varying the frequency sweep interval. In this study, a sweep interval of 2.5 kHz is used. Given the resonance method’s calibration coefficient of 5.05 MPa/kHz for the M5 × 10 bolts, the stress resolution is 12.63 MPa.

Despite the higher stress resolution of the time delay method, the actual measurement accuracy is influenced by the signal strength and signal-to-noise ratio (SNR) of the reflected echoes. Consequently, the resonance method achieves higher measurement accuracy. This further demonstrates the superiority of the resonance method in measuring the pretension of small-sized bolts.

### 5.3. Measurement Error Comparison

**Figure 12:**
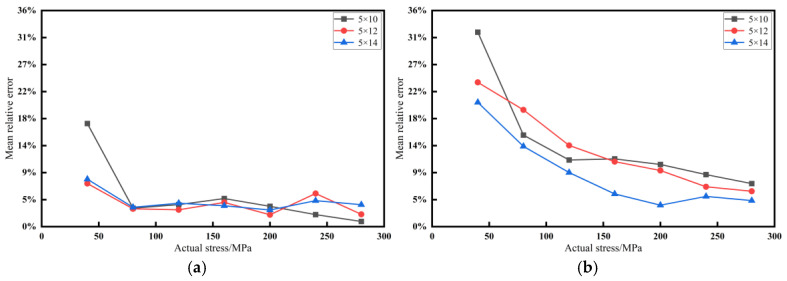
Average relative error curve of each sample: (**a**) resonance method; and (**b**) time delay method.

To more intuitively evaluate the measurement accuracy of the piezoelectric ultrasonic resonance method and the time delay method, an error analysis is conducted for each bolt specification. The average relative error from three measurements ias used to plot the error curves, as shown in Figure 12. The overall error statistics are summarized in Table 15.

From the above data analysis, it can be observed that the resonance method maintains an overall accuracy of over 95% when measuring three specifications of M5 small-sized bolts, with minimal error fluctuation. In contrast, the time delay method exhibits lower overall measurement accuracy for these three specifications, with the highest average relative error reaching 13.75%. Additionally, the measurement accuracy of the time delay method gradually increases with the increase in bolt pretension. This phenomenon occurs because small-sized bolts only produce a sufficiently large elongation when subjected to higher pretension, making the time variation of the sound wave more noticeable.

In such cases, the resonance method, which has a high-frequency shift resolution, can accurately measure even the minute elongation changes in bolts under low pretension, thus avoiding significant accuracy fluctuations.

## 6. Conclusions

Combining acoustoelastic theory, ultrasonic resonance theory, and the stress–strain model of bolts, a measurement model for the pretension of small-sized bolts based on the ultrasonic resonance method is proposed. The relationship between ultrasonic resonance frequency shift and bolt pretension is theoretically derived and experimentally validated. The following conclusions can be drawn from the experimental results:

(1) Within the yield stress range of the bolt, the ultrasonic resonance frequency shift is approximately linearly related to the bolt pretension. The frequency shift coefficient can be obtained through calibration experiments.
(2) The resonance method outperforms the traditional time delay method in three critical parameters: ultrasonic signal strength, repeatability and consistency of the fitting curve, and measurement error. Therefore, the ultrasonic resonance method is more suitable for measuring the pretension of small-sized bolts.
(3) For the three specifications of bolts, the proposed ultrasonic resonance method has a maximum measurement error of approximately 17.18%, with the average measurement error being within 5%. In comparison, the traditional time delay method has a maximum measurement error of about 32.38% and a highest average relative error of 13.75%, demonstrating superior measurement accuracy.

## Figures and Tables

**Table 1:** Experimental equipment.

Equipment Name	Equipment Model	Main Parameters
Tensile testing machine	HDW-50K, Ling, China.	Measurement accuracy: 0.5% Application range: 0~100 kN
Piezoelectric transducer	PZT-5H, Shenra, China.	Diameter: 5 mm Center frequency: 10 MHz
Oscilloscope	MDO3034, Tektronix, Beaverton, OR, USA.	Sampling rate: 2.5 GS/s Max recording range: 10 M
High-energy ultrasonic system	RAM-5000 SNAP, Ritec, Rochester, NY, USA.	Max excitation power: 5 kW

**Table 2:** Bolt parameters.

Specimen	Diameter (mm)	Total Length of Bolt (mm)
A	M5	10
B	M5	12
C	M5	14

**Table 3:** Fixed parameter settings for the RITEC experimental system.

Parameters	Value	Parameters	Value
Start frequency	9 MHz	Gain	22 dB
Stop frequency	11 MHz	Integr. rate	348 V/Vms
Increment frequency	2.5 kHz	Gate width	10 μs
Low pass	20 MHz	High pass	4 MHz

**Table 4:** RITEC test system sets the bolt parameters for different specifications.

M5 × 10		M5 × 12		M5 × 14	
Parameters	Value	Parameters	Value	Parameters	Value
Out level	13	Out level	25	Out level	35
Burst width	9.6 μs	Burst width	10.4 μs	Burst width	12 μs
Gate delay	14.3 μs	Gate delay	15.8 μs	Gate delay	18 μs

**Table 5:** The frequency shift coefficient obtained by calibration.

Specimen	Clamping Length (mm)	Serial Number	$\mathrm{Frequency}\;\mathrm{Shift}\;\mathrm{Coefficient}\;K_{R}$ (MPa/kHz)	$R^{2}$
A	5	1	5.08	0.9955
		2	4.88	0.9918
		3	4.81	0.9921
		4	5.21	0.9965
		5	5.25	0.9903
		Mean value	5.05	---
B	6	1	4.77	0.9979
		2	4.79	0.9978
		3	4.67	0.9974
		4	4.83	0.9997
		5	5.00	0.9955
		Mean value	4.81	---
C	7	1	4.68	0.9999
		2	4.65	0.9925
		3	4.46	0.9989
		4	4.47	0.9990
		5	4.43	0.9992
		Mean value	4.54	---

**Table 6:** Sample A measurement results of the ultrasonic resonance method.

Actual Stress (MPa)	Serial Number		
	#1 Measured (MPa)	#2 Measured (MPa)	#3 Measured (MPa)
40	32.43	46.83	33.79
80	76.40	77.66	81.45
120	125.64	114.28	121.85
160	172.35	153.41	163.51
200	211.49	193.81	202.65
240	246.84	234.21	241.79
280	280.93	274.61	280.93

**Table 7:** Sample B measurement results of the ultrasonic resonance method.

Actual Stress (MPa)	Serial Number		
	#1 Measured (MPa)	#2 Measured (MPa)	#3 Measured (MPa)
40	37.28	43.72	42.18
80	81.77	82.64	77.27
120	125.06	116.53	118.42
160	148.73	158.71	166.79
200	206.83	202.49	197.41
240	233.30	256.71	256.31
280	274.18	277.21	288.66

**Table 8:** Sample C measurement results of the ultrasonic resonance method.

Actual Stress (MPa)	Serial Number		
	#1 Measured (MPa)	#2 Measured (MPa)	#3 Measured (MPa)
40	37.45	43.72	36.78
80	83.99	78.41	77.84
120	122.58	125.59	114.12
160	158.90	165.43	149.81
200	195.22	205.35	193.47
240	232.67	246.19	257.67
280	271.45	294.62	287.58

**Table 9:** The relative measurement error of three samples by the resonance method.

Actual Stress (MPa)	Sample		
	A Measurement Error Δ	B Measurement Error Δ	C Measurement Error Δ
40	17.18%	7.18%	7.91%
80	3.08%	2.98%	3.23%
120	3.67%	2.81%	3.90%
160	4.68%	4.03%	3.48%
200	3.39%	1.99%	2.78%
240	2.00%	5.52%	4.33%
280	0.86%	2.06%	3.66%
Mean value	4.98%	3.79%	4.18%

**Table 10:** Calibration results of the pretension coefficient determined by the time delay method.

Bolts Size	Serial Number	$\mathrm{Coefficient}\;K_{L}$ (MPa/ns)	$R^{2}$
M5 × 10	1	11.40	0.9996
	2	11.06	0.9993
	3	11.83	0.9986
	4	12.03	0.9976
	5	11.31	0.9994
	Mean value	11.53	---
M5 × 12	1	9.24	0.9996
	2	8.85	0.9980
	3	9.07	0.9989
	4	9.16	0.9986
	5	8.43	0.9998
	Mean value	8.95	---
M5 × 14	1	7.62	0.9974
	2	7.53	0.9992
	3	7.27	0.9989
	4	7.43	0.9998
	5	7.29	0.9996
	Mean value	7.43	---

**Table 11:** Sample A measurement results of the ultrasonic time delay method.

Actual Stress (MPa)	Serial Number		
	#1 Measured (MPa)	#2 Measured (MPa)	#3 Measured (MPa)
40	29.52	50.23	21.85
80	66.91	69.56	66.91
120	105.15	132.53	107.43
160	142.48	178.68	142.02
200	179.35	180.55	177.98
240	217.13	221.51	218.95
280	255.82	265.84	258.10

**Table 12:** Sample B measurement results of the ultrasonic time delay method.

Actual Stress (MPa)	Serial Number		
	#1 Measured (MPa)	#2 Measured (MPa)	#3 Measured (MPa)
40	29.92	50.48	48.29
80	67.30	101.76	92.24
120	106.83	100.44	135.97
160	141.72	177.10	143.41
200	182.31	215.29	176.90
240	228.76	254.59	262.02
280	264.47	301.29	292.77

**Table 13:** Sample C measurement results of the ultrasonic time delay method.

Actual Stress (MPa)	Serial Number		
	#1 Measured (MPa)	#2 Measured (MPa)	#3 Measured (MPa)
40	28.83	32.49	46.15
80	67.17	88.67	90.58
120	108.34	131.07	129.73
160	154.54	170.00	170.70
200	196.45	208.93	208.94
240	226.47	249.78	252.90
280	264.21	290.75	289.96

**Table 14:** The relative measurement error of three samples determined by the time delay method.

Actual Stress (MPa)	Sample		
	A Measurement Error Δ	B Measurement Error Δ	C Measurement Error Δ
40	32.38%	24.04%	20.69%
80	15.26%	19.46%	13.37%
120	11.10%	13.53%	9.02%
160	11.29%	10.83%	5.45%
200	10.35%	9.35%	3.57%
240	8.67%	6.65%	5.03%
280	7.17%	5.90%	4.35%
Mean value	13.75%	12.82%	8.78%

**Table 15:** Overall mean relative error.

Sample	Resonance Method Mean Relative Error	Average Relative Error of Time Delay Method
M5 × 10	4.98%	13.75%
M5 × 12	3.79%	12.82%
M5 × 14	4.58%	8.78%

## Data Availability

The original contributions presented in this study are included in the article. Further inquiries can be directed to the corresponding author.
